# A Treatment Plan for Dogs (*Canis familiaris*) That Show Impaired Social Functioning towards Their Owners

**DOI:** 10.3390/ani10010161

**Published:** 2020-01-17

**Authors:** Joke Monteny, Christel Palmyre Henri Moons

**Affiliations:** 1 Department of Biotechnology, Vives University College, 8800 Roeselare, Belgium; 2 Ethology and Animal Welfare Research Group, Department of Nutrition, Genetics and Ethology, Ghent University, 9800 Merelbeke, Belgium; Christel.Moons@UGent.be

**Keywords:** *Canis familiaris*, dog, impaired social functioning, fear, anxiety, desensitisation, cue-induced relaxation, classical conditioning

## Abstract

**Simple Summary:**

Many domestic dogs are uncomfortable when humans perform everyday actions because the animals perceive them as threatening. One way to treat this is to expose a dog to the problematic stimulus with increasing intensity while the dog remains relaxed, resulting in desensitization. However, when it is the owner’s actions that the dog feels threatened by, it is difficult to apply such treatment. We present a programme for dogs with impaired social functioning towards the owner, consisting of (1) increasing owner knowledge and awareness regarding dog body language and perception of owner actions, (2) management of everyday life with the dog through general stress reduction and avoidance of stressful situations, and (3) behaviour modification through training. We also describe five cases to examine the results that can be obtained using this programme and propose future research.

**Abstract:**

Many domestic dogs are uncomfortable when humans perform trivial and benign actions that the animals perceive as threatening. A common technique for addressing canine emotional discomfort involves desensitization, where the intensity of a problematic stimulus is gradually increased while the dog remains relaxed. Desensitization requires a skillful owner and is complicated when actions of the owner are the stimuli to be desensitised. This paper introduces a behaviour modification programme for dogs with impaired social functioning in relation to the (inter)actions by their owners, consisting of (1) increasing owner knowledge and awareness regarding dog body language and perception of owner actions, (2) management of the daily life of the dog through general stress reduction and avoidance of stressful situations, and (3) behaviour modification through training. The latter component entails a non-threatening, predictable exercise in which the dog has control over any perceived threats, the introduction of the safety cue with subsequent desensitization, and engaging activities with the owner that the dog finds enjoyable. We also present a case series report to examine a selection of dogs with impaired social functioning, from signalment to outcome, when treated with the proposed behaviour modification and examine which adaptations were made to the plan according to individual dogs. Finally, we avenues for future research.

## 1. Introduction

Many dogs show impaired social functioning, i.e., the interaction with and actions by humans are problematic. This, in turn, may lead to fear and anxiety in the presence of human stimuli. Drawing from the accepted behaviouristic definitions of fear and anxiety, fear towards humans is short in duration, stimulated by the action of a person and ends when the action stops, whereas anxiety is a prolonged condition in which the dog expects (inter)actions from the owner and anticipates the associated discomfort, or continues to anticipate it even after an interaction has ended [1,2,3]. A dog being uncomfortable around humans poses a risk for the wellbeing of both the human and the dog. Humans may be faced with disruptive behaviour by the dog and they can become the victim of injurious dog aggression [4,5,6]. Dogs, on the other hand, suffer from chronic stress when uncomfortable around humans. Furthermore, the behaviour and behavioural problems they develop could, in turn, lead to a breakdown in the bond between the owner and the dog, possibly resulting in relinquishment or even euthanasia [7,8,9,10,11,12].

Impaired social functioning in dogs is not uncommon, although it is difficult to extract exact numbers from literature due to issues of terminology and the use of diagnostic categories that do not allow us to identify the underlying emotion of a behaviour [13,14]. Alternatively, anxiety disorders are often mentioned as part of behavioural problems, but most often—and with the exception of separation anxiety—they are grouped under a general category of “anxiety” rather than related to the presence of social or non-social stimuli [15,16,17]. For fear, the subdivision of social and non-social is made, but this generally pertains to unfamiliar people or dogs [18,19].

Impaired functioning in relation to particular stimuli or contexts can be managed in different ways. Older methods involve the use of flooding and positive punishment, the former intended to alter the emotion towards the issue and the latter to reduce the occurrence of behaviours resulting from the emotion [20]. Both involve risks for the welfare of the dogs and the people applying the methods. More recently, less confrontational methods are increasingly advocated, such as the use of medication, management of the environment (e.g., avoiding triggers), application of a desensitization and/or counterconditioning protocol, or a combination thereof. Medication may be useful for anxious dogs to stimulate their memory and increase their ability to learn [21,22,23,24]. Managing the environment is important to reduce aggressive behaviour, and it can also be used to reduce anxiety [25,26]. Desensitization is a popular technique for addressing problematic stimuli in the dog [27,28]. During a desensitization program, the dog is exposed to a stimulus at an intensity that does not elicit a response. Next, the intensity is gradually increased while the dog remains relaxed. Desensitization can be used in combination with counterconditioning, whereby the presentation of the stimulus is paired with something the dog finds pleasant—e.g., food. Desensitization requires a skillful owner and is complicated when actions of the owner are the stimuli to be desensitised. When even minimal actions already trigger an emotional response in the dog, it is difficult to find a starting point—i.e., a relaxed dog.

Several methods for relaxation can be found in literature. First, relaxation can be induced in dogs using various techniques. First, there are methods based on physical contact by the owner, such as massage, posture facilitated relaxation, relaxation soft method, Tellinghon TTouch methods, or pressure on the body using body wraps or a ThunderShirt^®^ [29,30,31,32,33]. Second, relaxation can be induced using operant procedures, where the animal is reinforced for showing relaxed behaviour [31]. Initially, the smallest physical sign of relaxation is reinforced, following which the owner can progress to more and then complete relaxation in the dog. It is then assumed that because the dog shows physical signs of relaxation, there is also a corresponding emotion. Finally, it is also possible to obtain relaxation by inducing an emotion of safety through classical conditioning [34,35]. Safety is defined in several ways, according to psychology literature (as reviewed by Andreatta and Pauli 2017), such as the disappearance of an ongoing threat (relief), the non-occurrence of an expected threat (respite), and the absence of threat [36]. Particularly the latter has been studied extensively in the paradigm of differential conditioning where an initially neutral stimulus (CS+) is paired with an aversive unconditioned stimulus (US), while another neutral stimulus (CS−) is never paired with the US. This CS− is also referred to as a safety signal (Van Damme et al. 2004). Such safety/relaxation cues may be verbal, non-verbal or physical. They are widely used in practice and this paper describes one form of application. The advantage of using this type of classical conditioning when inducing relaxation in the dog is that it is a passive process from the perspective of the animal conducive to relaxation, it requires no action from the owner that could be perceived as threatening by the dog and it requires less technical skills from the owner compared to operant conditioning.

This paper introduces a treatment plan for dogs showing impaired social functioning in relation to the (inter)actions by their owners. We also present a case series report to examine a selection of dogs with impaired social functioning, from signalment to outcome, when treated with the proposed behaviour modification and examine which adaptations were made to the plan according to individual dogs. Finally, we suggest avenues for further research into the mechanisms of the proposed treatment programme.

## 2. Behaviour Modification Plan

The treatment consists of three main components: increasing owner knowledge and awareness, management of the daily life of the dog, and behaviour modification through training. For ease of reading, the text refers to owners and dogs as being male throughout; but female is implied as well.

### 2.1. Increasing Owner Knowledge and Awareness

#### 2.1.1. Developing Knowledge and Skills Regarding Observation and Interpretation of Dog Body Language

Owners receive theoretical information about dog communication and body language. They are informed about which social stress signals their dog shows, using the Ladder of Aggression by Kendall Shepherd as a guide [37].

While with the counsellor, short videos are made of the owner performing benign actions in the presence of the dog (standing up, walking around, putting the leash on, and petting). The owner is also asked to make some videos in the home environment of situations in which the owner believes that the dog is relaxed. Coached by the counsellor, the owner practices observing dog behaviour and interpreting the emotional state of the dog and its evolution over time, taking into account the context in which the dog shows the behaviour.

The skills of observing and interpreting dog behaviour are further developed during the “predictability game”, as explained below.

This part of the Behaviour Modification Plan (BMP) is important because being able to observe and correctly interpret dog body language are key for successfully going through all steps of the BMP [38,39]. Just being around dogs does not necessarily result in such knowledge and skill [40]. In addition, dog body language knowledge is not only an important part of canine aggression prevention, but it is also crucial for a good human–dog relationship [41].

#### 2.1.2. Understanding the Fact That the Dog Is Socially Impaired

The counsellor provides insight in the subjective experience and appreciation by the dog of specific events and situations and how this may differ from the owner’s perspective. An owner may intend to show affection to the dog by performing behaviours that he would also do towards another person, but the dog may experience these actions as threatening. The owner must also fully understand which coping strategy or strategies—and corresponding behaviours—their dog prefers to use during situations of perceived stress in an attempt to gain control over the situation. Possible coping behaviours are mounting, initiating and insisting on playing, and displaying what appear to the owner as attention-seeking behaviours (e.g., putting his paw on the knee of the owner, pushing his nose under the owner’s arm, obsessively bringing toys, barking at the owner, destroying items, insisting on being petted)

The counsellor creates two settings, in consecution. One is a setting without interaction with the dog, and the other involves actions towards the dog by the counsellor (these are benign and trivial actions like looking at the dog without staring or walking in the direction of the dog). Each setting lasts about one minute and the counsellor points out relevant behaviours to the owner or records the dog on video and discusses it afterwards. Comparing the behaviour of the dog in those two different situations helps the owner understand the perception of the dog. It is important to mention to the owner that there has been no previous learning process between the dog and the counsellor, so that the behaviour is a spontaneous response of the dog to trivial actions and not a conditioned response to the person performing them.

This part of the BMP is relevant to help the owner understand the key problem in the dog, i.e., the difficulty that the dog has with human actions, even those without physical contact.

#### 2.1.3. Owner Awareness of Own Actions towards the Dog

As a final aspect of this component of the BMP, the counsellor makes the owner aware of his own actions, how the dog most likely perceives these actions, and how it impacts the stress level of the dog. To avoid guilt in the owner, however, it is important to stress that the owner’s actions are not the cause of the issue.

This part of the BMP is included as a means to give the owner more insight and to prepare him for Section 2.2 of the BMP.

### 2.2. Managing the Daily Life of the Dog

#### 2.2.1. General Stress Reduction

Using information provided by the owner, all situations in the daily life of the dog where it seems that he is uncomfortable are discussed. As the owner initially may not be aware of all situations, more can be identified during the course of treatment. Such situations are typically moving about while the dog is near, feeding the dog, putting a collar or harness on the dog, going for walks, and playing with the dog. The counsellor encourages the owner to think about which practical interventions are feasible to improve the situation for the dog, depending on the specific issues and home context. Examples of interventions include changing the location of the crate, using barriers, giving the dog food in another place, changes to the walking routine (where and how) or play routine (which kind of play and how to play). Owners are also trained to anticipate problems during everyday life with the dog and to identify and apply solutions to prevent problem situations arising.

This is an important aspect of the BMP because stress can affect learning abilities, which could adversely affect the success of the BMP [42,43]. Also, anxiety and stress were found to have a negative effect on dog welfare, health and lifespan [44,45].

#### 2.2.2. Avoiding Stressful Situations with the Owner and the Use of Positive Punishment

Even in a modified version, some activities with or towards the dog cannot be maintained because of the problematic actions involved. Such activities should be avoided altogether, although usually only temporarily. Together with the owner, the counsellor searches for alternative solutions.

In case the owner uses positive punishment for unwanted behaviours shown by the dog (e.g., verbal reprimand, yelling, pushing the dog, jerking the leash, staring at the dog, taking the dog by the collar), the counsellor explains why it should be avoided in future. The situations where the owner uses positive punishment are discussed and the counsellor helps the owner to find alternative solutions, aimed at avoiding the behaviour or deterring it, the owner responding differently to it and/or differentially reinforcing other behaviour (e.g., walking away while calling the dog to him and asking for another behaviour).

The rationale for this aspect of the BMP is that exposing the dog to stressful situations may create and/or strengthen the negative association with the presence of the owner, which is what the BMP tries to avoid and/or solve. In addition, when in stressful situations, a dog may show unwanted and sometimes dangerous behaviour. When the owner uses confrontational techniques such as positive punishment to counter these behaviours, there is a risk of escalation of the problem and aggression by the dog [46].

### 2.3. Behaviour Modification through Training

#### 2.3.1. The Predictability Game

The owner performs this exercise at home, in an area of the house where the dog is most likely to be comfortable and has the chance to distance himself from the owner if he so chooses. The exercise begins with the owner announcing the start using a pre-determined cue, like “start exercise” and takes then a limited amount of small-sized kibble or treats (5 to 15 pieces). The kibble should not be of such high value to the dog that it elicits arousal. The owner says “take it”, waits two seconds, and then throws one treat on the ground. After a few seconds, the procedure of saying “take-it”, waiting two seconds and throwing a treat on the ground is repeated. The dog is free to take the treat or leave it. The owner is instructed to avoid direct eye contact while observing his/her dog. Although eye contact in many cases is the start of every human–dog interaction (Miklosi et al., 2005), it could also be interpreted by dogs as a threatening signal. The owner continues the procedure even if the dog is not eating the pieces of kibble immediately. When all the treats are thrown, the end of the training is announced, always using the same word and the owner walks away while ignoring the dog.

The rationale for including this exercise in the BMP is to create a situation for the dog in which the actions of the owner are fully predictable and in which he has control over perceived threats. This situation cannot be created from actions in daily life as most of those already elicit negative emotions in the dog. Predictability is created by announcing the start and end of the exercise using a verbal cue and the fact that the owner behaves in a predictable way without any actions directed at the dog. The dog has control because he is free to choose at which distance he will be from the owner at any time. He is also free to take the treat or leave it: throwing the treat and the action of the dog are independent as such. The treat is merely intended to give the owner something to do with and for the dog and, in case the dog eats the treat, it is something nice for the dog, which may induce the formation of a classically conditioned association. Over time, the predictability game becomes a context in which the dog can correctly anticipate the owner’s actions, which helps the dog feel safe. This exercise, once the dog has learnt it, can also be used to defuse an accidental everyday situation that the dog perceives as threatening. In addition, this exercise further trains the owner to observe and interpret dog body language.

The exercise is considered to be successful if the following criteria are met: the dog is emotionally able to participate in the exercise, the dog remains near the owner during the exercise, the dog is engaged without being hyperactive or showing other signs of discomfort (e.g., panting, trembling, lifting his paw, barking), the dog eats the kibble when the owner throws it, and the dog is able to cope when the owner terminates the exercise. As a learning tool for the owner, this exercise is successful when the owner becomes proficient at recognizing body language expressed by the dog, particularly the identification of (subtle) signs of stress, which are likely to occur in the early stages of performing the exercise.

#### 2.3.2. Classically Conditioning a Safety Cue

The purpose is to classically condition a cue (CS-, “safety cue”) that signifies nothing threatening will happen as long as the cue is present. This training takes place at home.

##### Identifying a Situation of Relaxation

The training starts from a known situation where the owner and the dog are in the same room and the dog appears relaxed and, therefore, feels safe. Such a situation is characterised by the fact that within minutes after being put in this situation and—without any commands given to the dog—the dog shows relaxation—i.e., the dog is lying down somewhere without scanning the environment or monitoring the owner, playing with toys or chewing. This situation must be able to be created without external interference (e.g., not at a time when the doorbell is likely to ring or the mailman will pass by) and without association with feeding, playing or another activity with the dog. In many cases, this will be a situation where the owner is static, i.e., the owner does not move and is standing or sitting. The owner has to search for at least one such situation and map it for possible stressors and/or distractors by answering a number of questions in order to create the optimal training conditions. Such questions are: Who of the owners is present with the dog in the same room? What is that person or are those persons doing? In which room and area of the room is he/are they? At what time of the day can this happen without external interference? Should additional measures be taken, such as closing shutters or curtains, turning on the radio, removing the ironing board? In case there are other pets, where are they at that moment? Can they be in the same room or should they be elsewhere? Before progressing to the conditioning of the safety cue, the owner must test whether the situation is sufficiently likely to induce relaxation. For this purpose, the situation is tested five times. The dog must relax within the first 5 min after the start of the trial for at least 4 out of 5 trials.

##### Introduction to the Safety Cue

The safety cue (CS−) is a novel item for the dog, without any previous association, for example, a mat. At the start of the training, the owner takes the mat and one piece of kibble. The owner makes sure that he and his dog are in the same room. The owner announces the fact that he will put the safety cue on the floor, such as “mat is here” and puts the mat on the floor together with one piece of kibble on it. Directly afterwards, the owner creates the above-mentioned relaxing starting situation. The dog may or may not take the kibble from the mat. The animal will experience the context of the absence of threatening actions by his owner and become relax. During 5 to 10 min, the mat lays on the floor, while the owner continues to perform no action toward the dog. This means that the owner occupies himself, e.g., by reading, and does not look at the dog, except for an occasional glance to observe if the dog is still relaxed. The owner then removes the mat. When the dog is not lying on the mat or nearby, the owner can easily remove the mat without disturbing the dog. When the dog is lying on the mat, the owner announces to the dog that he will remove the mat by calling the dog in his direction. He then moves away from the dog and goes to get the mat without going in the direction of the dog.

##### Checking the Significance of the Safety Cue

The process of CS− conditioning is repeated over the course of several days or weeks, until the dog has made an association between the safety cue and the behaviour of the owner (absence of threat), resulting in the emotionally conditioned response of feeling safe, secure and relax. The number of required repetitions depends on the learning abilities of the dog and the available time of the owner. The installation of the conditioned emotional response can be observed when the dog becomes relax upon noticing the cue. Being fully relaxed may take up to a few minutes. Relaxation is evaluated using the following observations: the dog is lying down, is not scanning the environment, has his eyes closed and is breathing calmly. The presence of an association is checked at a different location (e.g., another room, or at the training facility) than where the training took place, and it should also be a location where there was no previous evidence of impaired social functioning of the dog towards his owner. If the dog relaxes at this location within 5 min after the start of the exercise, and this in at least 4 out of 5 different repetitions, then the owner can proceed to the next step.

##### Progressing with Systematic Desensitization

The next step is to expand the initial training setting to a context where the actions of the owner were previously associated with negative feelings of the dog and, therefore, stress coping behaviours were seen. The stimuli used at first must be mild, e.g., the training situation can evolve from a static owner in the initial training setting to an owner moving around for a couple of seconds and then assuming the static position again. There is a chance that mild arousal or the manifestation of a coping strategy occurs in the dog when progressing. By then, however, the owner will have learnt to recognise these signs and be mindful of them while continuing not to do any actions towards the dog. This allows the vicious cycle of action of the owner, followed by the action of the dog and then the interaction of the owner to the dog to be broken. According to the progress made by the dog, the animal is exposed to—from the dog’s perspective—increasingly threatening actions in the presence of the CS−. The owner has to be aware of signs of stress and only if the dog consistently can become relax within the first 5 min, the setting can be made more challenging and more resembling of the problematic daily activities. Depending on the individual dog and the issues that were experienced, this training can also be expanded to other rooms in the house. A list of problem situations and contexts for the dog is compiled and prioritized by increasing perceived stress levels. These situations are successively associated with the presence of the safety signal in such a way that the intensity of the response by the animal is acceptable and that the animal is able to relax each time.

The rationale for conditioning the safety cue is to create a starting point for systematic desensitisation [47,48]. Classically conditioning such a cue enhances predictability for the dog regarding the absence of threat.

#### 2.3.3. Activities with the Owner That the Dog Finds Enjoyable

In order to create a positive association for the dog with the presence of the owner, the counsellor helps the owner identify activities with the dog that the animal would enjoy doing. This could be going for a walk on a long lead, during which the dog gets ample sniffing time, but it could also be sessions in which the owner trains a behaviour in the dog using positive reinforcement. Such sessions are intended as quality time with the owner, so not only should the dog experience the session as non-threatening, the animal should also enjoy it. It should also be noted that, unlike the predictability game that involves classical conditioning, these training sessions are about operant conditioning.

Whereas other aspects of the BMP focus first and foremost on avoiding negative emotions, this aspect of the BMP is intended to promote positive welfare in the dog and to associate the positive emotions with the presence of the owner as a means to restore the human–dog relationship [49,50,51].

#### 2.3.4. The Exercises Working Together in Practice

The predictability game (Section 2.3.1), mapping or developing a situation in which the dog feels safe (Section 2.3.2) and managing the daily life of the dog (Section 2.2) are the first steps. Once the situation in which the dog is known to relax has been identified or created (first step of Section 2.3.2), the safety cue (CS-) training can begin. The predictability game can be used to facilitate this training or other interactions with the dog, such as the training sessions mentioned in Section 2.3.3. The highly predictable setting of the predictability game is reassuring for the dog and brings the dog in a frame of mind that allows learning. In case of training the safety cue, for example, the owner starts the procedure with the predictability game and then finishes by presenting the CS−.

It is important to remember that all the exercises should be trained below the threshold of the dog for fear or anxiety. Once the dog is well-trained for the exercises, the owner can use them as predictable interactions to interrupt, change or prevent stress, and consequently prevent or deter unwanted behaviour of the dog.

## 3. Case Series Report

The purpose of the case series report is to examine a selection of dogs with impaired social functioning, from signalment to outcome, when treated with the behaviour modification plan described above. We also examine which adaptations were made to the plan according to individual dogs.

### 3.1. Case Selection

Out of 261 cases presented for behaviour evaluation and modification between 1 January 2017 and 1 January 2019 at the dog behaviour and training facility of the first author, five cases of dogs with socially impaired functioning towards their owners and for which the entire BMP had been applied were selected. Other inclusion criteria were

- The owners provided a fully completed questionnaire prior to the first consultation.
- The owners attended at least three sessions of coaching (including a first session with behaviour history taking) over a period of at least three months.
- The dog was healthy and was seen by a vet, at least one month before the first session.
- The exclusion criteria were
- Indication of abuse of the dog by the owner.
- Dogs given psychotropic drugs during the treatment.

All dogs also showed impaired social functioning to unfamiliar people, but this was not an inclusion or exclusion criterion, and this issue is not the focus of the current BMP.

### 3.2. Case Presentation

The dogs ranged in age from 7 months to 5 years and were diverse regarding breed, gender, neutering status, origin and living conditions (Table 1). One male dog (D) was neutered at the age of about 9 months, following the complaint of barking at other dogs and subsequent advice by the veterinarian to neuter.

The presenting complaints from the owners were also multiple and diverse: pulling on the leash during walks, being extremely active, being extremely calm, showing dominant behaviour towards the owner, being aggressive towards the owner and/or strangers, showing aggression towards other dogs, being disobedient, being anxious, stealing objects, and eating objects.

### 3.3. History

All dogs were acquired by the owner at 7–9 weeks of age, except for dog E, which had been bought at 7 months of age.

Three owners (of dog A, C, and D) attended group puppy classes at a dog school soon after the puppy arrived at their home. The classes focused on obedience training, such as teaching the dog a sit, down, recall, or to walk on a loose leash. Upon further investigation, it became clear that several problem behaviours were already present at that time. These problems were not related to the dogs learning new tricks, but to how they behaved in general during the classes.

Dog D had a history of snap and bite incidents, which started at very young age (about 10 weeks). Victims were both familiar people—the owner or the daughter who visited—and passing strangers on walks. In all circumstances, the actions of the humans were benign from a human perspective and/or not directed at the dog.

All owners reported that their dog was highly active, except for dog E. From the moment the owners had dog E, the animal had shown extreme inhibition (freezing) to the actions of some family members, such as going in the direction of the dog, touching or petting the dog. Towards other family members, the dog growled, barked and showed bite threats. Luring the dog closer to those family members with food did not improve the behaviour of the dog.

As the dogs became older, their owners (except for the owner of dog E) classified them as ‘attention seeking’. The owners described this as the dogs approaching them and pawing at their arm or pushing against them with their muzzle. They also described behaviour like jumping up, barking at the owners or insisting on play by bringing toys and continuously following the owner around the house. All dogs, except dog E, were obsessively following their owner while the owners were moving around in their house.

All owners had tried several solutions to change the behaviour of their dog. The solutions were given to them by dog trainers, veterinarians, or they came up with them themselves. Such solutions were: giving the dog more activity by playing more with the dog or increasing the duration of the walks; giving the dog more distraction such as chewing toys, activity balls, brain and scent work; attending obedience classes; applying the “nothing is in life is free” method, or neutering the dog.

### 3.4. Behaviour Assessment

For all dogs, the first session at the facility was a 120-min behaviour consultation, comprised of a behaviour assessment and initiation of the BMP. The behaviour assessment consisted of a written questionnaire completed by the owner prior to the first visit to the facility, an interview with the owner and a behavioural observation. In the questionnaire, information about the general behaviour of the dog, the onset and evolution of problem behaviour, and the reaction of the owner were examined. During the consultation, the behaviour of the dog, the interaction between the dog and his owner, and the difference in response to trivial actions by the owner and the counsellor were observed.

### 3.5. Diagnosis of Socially Impaired Functioning towards the Owners

The behaviour assessment revealed a systematic link between actions by the owners and the emotions of and behaviour by the dogs. All dogs showed clear signs of discomfort in response to or anticipation of trivial, benign actions of their owner, such as looking at the dog, approaching or passing the dog, leaning over the dog, and petting the dog. Stress signals ranged from lowering the ears, lip licking, panting, turning head and body away, paw lifting, sitting, going away to snapping, growling, and severe inhibition. Other observed behaviours were stealing objects, barking, jumping up, obsessive following and scanning the owners, restlessness, continuous walking around, disobedience, hyperactivity, growling, snapping and biting to the owners, tearing clothes, barking towards the owners, mounting and mouthing the owners, obsessive ball playing, excessive licking of legs and/or face of the owners, fleeing away when going to the dog, inhibition behaviour, obsessive chewing on toys and objects. The more subtle signs of emotional discomfort emitted by the dogs were not detected by the owners. The coping strategies displayed by the dogs differed somewhat between them, but they were all elicited by activity from the owners and—during the consultation—by the counsellor. In all cases, the dog was able to relax more when the owner(s) or the counsellor was/were not performing any action towards the dog.

Based on the information provided in the questionnaire, the interview and the observations by the counsellor during the behaviour evaluation, the counsellor found all dogs were suffering from socially impaired functioning in relation to their owners [19].

### 3.6. Treatment

For all cases, the BMP as described above was initiated during the first session. More specifically, the owner was given information about dog body language and was made aware of the problem for the dog (Section 2.1). The management of the daily life of the dog was examined and discussed (Section 2.2). These aspects were revisited during follow-up sessions, if the owner needed to be reminded or if some daily-life situations needed to be managed differently as the treatment progressed. The predictability game (Section 2.3.1) was also introduced in the first session and, if appropriate for the dog, practised. Finally, the counsellor helped the owner identify a situation of relaxation at home (first step of Section 2.3.2).

With regards to management of the daily life of the dog, many recommendations were similar for all dogs, such as advice for walking (avoiding walks on short leash or in busy areas where the dog cannot keep at a distance), showing affection (avoiding physical contact like hugging, but also staring at the dog) and managing visits from unfamiliar people (placing the dog in a room where he cannot see the visitors). For dog A and B, additional advice was refraining from rough play with the dog with all members of the family. Additionally, owner A was advised to temporarily stop walking with the dog and instead give the animal exercise in the garden. For dog B, the owner was asked to let the dog choose when he wanted to stay in the garden if she went inside instead of calling him in. The owner of dog C was asked to stop daily obedience training with the dog. For dog D, the interaction with his ball and the owner was made more structural. So they would play with the ball only on discrete moments of the day, and with only a discrete number of interactions, such as the owner would throw the ball three times and then put away the ball even when the dog would ask for more.

All dogs, except dog E, were immediately engaged in the predictability game (Section 2.3.1). At the beginning of the exercise, most dogs (A, B, C, and D) would jump against the owner. This was remedied using negative punishment. Subsequently, all dogs learned the exercise after a maximum of 5 repetitions, as evaluated by the criteria described above. For dog E, it took more time before the exercise was successful as he was unable to stay engaged. Dog A and B had difficulties coping with the end of the exercise: they expressed behaviour such as jumping up, biting, and stealing an object. As a result, the end of the exercise was modified: after announcing the end of the exercise, the owner gave the dog something to chew on and guided the dog to his crate. This resolved the issue by the next session at the facility (one month later).

The questionnaire and the interview were used to identify a situation at the owner’s home in which the dog could relax (first step of Section 2.3.2). If the owner thought a particular situation would be suitable, the counsellor asked the owner to record video footage of the dog in that situation to confirm relaxation. If the relaxation could not be confirmed, another situation had to be tested in a similar way until an appropriate situation was found. For dogs A and C, this context consisted of the owner working on the computer and for dogs B and E, the owner would be reading a book at the kitchen table or on the sofa. For dog D, many situations had to be tested, mainly because the owner was unable to correctly interpret the body language of the dog and mistakenly identified the dog as being relaxed. Finally, the counsellor decided to create a new context in which the owner was asked to completely ignore the dog. Specifically, the owner was instructed to sit at a table where he/she had not sat before.

The introduction and conditioning of the safety cue was done at the owner’s home. Because the social impairment was not limited to one person but to all members of the household in all cases, it was necessary that either only one person was present during the training or that all persons present acted in a non-threatening way from the perspective of the dog. For dog A, C and D, the appropriate context at first was that only the owner who came to the facility was present during the introduction of the safety cue. For dogs B and E, two family members were involved with the training and either they trained the dog alone, or they were both present and acted in congruence with each other. For dog E, one particular family member (the father of the owners who lived with them) could not be present. Table 2 shows the timing of the training of the safety cue and the situation in which this occurred.

Once the safety cue had been conditioned, more situations of increasing emotional difficulty for the dog were successively desensitised. These were evaluated and discussed during follow-up sessions. For dog A, the subsequently trained situations were a situation identical to the starting situation in the home office, but now the owner moved during part of the time the safety cue was present. Next, the same two settings (static and moving) were trained in the kitchen, followed by a setting where the owner takes an object from the counter and puts it back, ending with the owner performing daily activities in the kitchen as well as in the dining room. The situation of the owner sitting on the couch was first desensitised during a time when normally the owner would not sit on the couch. Next, a situation where the other family member also sat on the couch was desensitised, ending with desensitization to both owners sitting on the couch in the evening.

For dog B, only the really challenging situations for the dog, where the behaviour of the dog also caused problems for the owners (barking, jumping up, stealing and destroying toys), were desensitised. The other situations, where the dog only followed or scanned continuously, were managed using other parts of the BMP. Situations which were desensitised were actions of the owner in the kitchen, first with the owner walking around a few steps and then sitting again, followed by a situation with continuous movement, then movement of short duration while the owner carried an object, ending with the owner performing everyday activities in the kitchen. The process was repeated for when a second person was present in these situations, but the dog now progressed quicker. Finally, also dining room activities and sitting on the couch were desensitised.

For dog D, the first focus was on the static position of one owner but at different locations and with different static activities such as ironing, reading, knitting. When the situation required preparation (setting up the ironing board), this was done while the dog was not in the same room. Situations that were desensitised were sitting down by the owner, then walking around (e.g., going to the ironing board), then performing an activity (e.g., ironing) but calmly and while standing still. For that dog, the presence of a second person also had to desensitised as well as the evening routine sitting at the couch.

For dog E, desensitisation began with the presence of both owners who trained the safety cue and this was done until the dog was comfortable with the presence of both of them during normal daily activities. For this purpose, not all situations had to be desensitised, as the behaviour of the dog in some contexts improved using the rest of the BMP. Next, the presence of the father as a static figure in another room (but visible to the dog) had to be desensitised. Finally, increasingly more action by the father was incorporated in the training.

Walking on a long leash (between 5 and 8 m) and in quiet areas was one example of an enjoyable activity with the owner (Section 2.3.3), but for most dogs (except dog A, for which walks were temporarily suspended), it was also part of overall stress reduction (Section 2.2.1). Consequently, this was initiated at the beginning of the BMP as well. Only after a few sessions, other positive activities (Section 2.3.3) such as training a behaviour using positive reinforcement was recommended. All dogs, except dog D and E, were trained to sit and stay. Dog D was trained to go to a mat. For dog E, during the first 2 months, only walking and the predictability game could be done without evoking obvious stress signals in the dog. After two months, using positive reinforcement and operant conditioning without any hand or verbal cue, the dog was trained to go to a mat. As the sessions progressed and the dog became more and more acquainted with these actions, basic scent games were performed for all dogs, except dog E.

### 3.7. Treatment Outcomes

For the evaluation of dog A, during the final session, the owner reported that the dog no longer showed restless behaviour in the house, nor was he jumping up at the owners. The behaviour of obsessively following the owner(s) had disappeared. The safety signal was not being used anymore but was kept in the home office. The dog often spontaneously visited this room. The problematic behaviour during the evening ritual (owners sitting on the couch) had disappeared and the dog settled down and rested while the owners watched tv or read a book. Even when they got up to fetch something to drink or use the bathroom, the dog could remain relaxed. The owner reported a considerable overall behavioural change in the dog while in the house, from being very active, stealing objects, jumping up against the owner to being a calm and relaxed dog. Walking on a short leash was still a problem, but walks on a long leash were enjoyable for both the dog and the owner. The arrival and presence of visitors was still an issue at that time, but it was managed by putting the dog in the home office, with the safety cue.

The evaluation at the final session revealed that dog B had stopped playing obsessively. Barking at the owners had also completely disappeared, and the dog was now resting most of the time while in the house. The safety signal was still used during the evenings and during several discrete situations, such as when the owner was cleaning the house. Walking on the long leash went well in familiar areas, but walking on a short leash was still an issue to work on.

The owner of dog C reported that the dog no longer obsessively followed him about the house and had almost entirely stopped showing—what she interpreted as—attention-seeking behaviour. Sometimes the dog was still showing such behaviour like pushing with his nose against the owner. When the owner ignored this behaviour, the dog soon stopped and could settle down. The dog had been walked 50% less than before the start of the BMP because the walks could only be performed in a quiet nature environment without other social contact and the owner had to take the car to reach such an area. Instead of walking her dog two to three times a day, the owner now did this three to four times a week. Even then the dog was much calmer. Visitors were still a problem but this was managed by putting the dog in an outside run. Walking on a short leash remained an issue.

From the start of the BMP until the final session, dog D had no more bite nor snap incidents (to the owner or strangers). The dog had several contexts in which he now was able to relax and no longer showed an obsession towards holding a ball in his mouth. Going out for a walk went well on a short leash.

Already 10 days after the safety cue was introduced, dog E was able to relax within 5 min of safety cue presentation when the owner remained static. After 6 sessions and more than six months of not being exposed to the father, the dog was able to relax in his crate in the presence of the safety cue and the father. No growling, barking or excitation occurred when the father was there. By the final session, the father could perform his daily activities when he was in the room with the dog and the dog was in the crate (door left open). The dog no longer showed behavioural inhibition while the owners were at home. The dog could also be walked on a long leash.

## 4. Discussion

This paper discusses a BMP for dogs that show social impairment (fear and/or anxiety in the presence of human stimuli) towards their owners. Using a case series, we have examined the application of this treatment plan to five dogs and investigated how the plan needed to be adjusted on an individual level.

There are some limitations to the case series. First, because the dogs were treated without the purpose of publishing their progress, we lacked sufficiently objective parameters to measure progress. For example, we are unsure of how many repetitions of the exercise were required for each dog to have successful conditioning of the safety cue. Second, we also lack quantitative data about the behavioural improvements in the dogs (e.g., frequency and duration of typical problematic behaviours, such as the dogs following the owner about the house). Third, we cannot be sure about the cause–effect association for each part of the BMP, mainly because of the limited number of cases and the lack of a case-control methodology. For example, the first author has already treated cases for which the safety cue was not required and now only uses the full BMP mainly when almost all daily life contexts are problematic for the dog. Of course, some aspects, like the effectiveness of increasing owner knowledge and the fact that relaxation improves learning, have already been demonstrated elsewhere [39,52].

The overall effect from the treatment that could be observed in the dogs from the case series was an increase in relaxation and less display of coping behaviours. We attribute this effect to the increase in—mainly—predictability and—also—control that is present throughout the treatment programme we propose [53]. Predictability has been indicated to be very important for other types of anxiety as well [34]. Other explanations for the observed effects could be habituation to the presence of people without any interaction with the dog as a result of the increased exposure to it during the treatment programme. However, it is known that habituation will lead to sensitisation if the stimulus intensity overtaxes the ability of the animal to cope and, in the cases we have reported, presence of the owners in many contexts was a problematic stimulus for the dogs. [54,55]. Another possible explanation is the fact that the dogs are aging. Age brings about several changes in behaviour, as discussed in the literature [56,57,58]. However, again, age in itself cannot explain fully the treatment outcome, as accumulated learning processes have not been found to be negated with age. On the contrary, they may fortify behavioural responses [58].

In the presented cases, the safety cue that was used by the owners was a mat, novel to the dog, so without pre-existing associations. Other visual cues can be used, such as a novel food bowl or a towel that is later reduced in size so it can be easily taken outside the home, and even auditory cues have proven to be effective in treatments applied by the first author. It is expected that a safety cue that is permanently present during the 5–10 min “no action” condition created by the owner will be easier to condition than an instantaneous cue. Also, in some situations, visual cues are more salient to dogs than verbal cues [59]. Research comparing the efficiency of different cue modalities (visual, olfactory, auditory) and the necessity of having a permanent cue versus whether an instantaneous cue would suffice, however, is currently lacking.

In principle, inducing relaxation is possible through operant or classical conditioning. In case of operant conditioning, this means the dog is reinforced for showing relaxed behaviour. A few potential problems can be identified with this technique, however. First, for dogs with social impairment, reinforcement is difficult because actions of the owner towards the dogs to provide the reinforcement (e.g., extending arm and reach toward the dog to give a treat) may be perceived by the dog as threatening. This is the same reason why techniques requiring physical contact (e.g., posture facilitated relaxation, soft relaxation exercise, massage and body wraps) would be problematic to use in dogs with social impairment. The need for action in order to reinforce could be circumvented by using a remote-controlled device to dispense treats. For the technique of cue-induced relaxation, however, the essence is that the owner does not do any action to the dog. Second, reinforcing relaxed behaviour requires an owner knowledgeable about dog body language, able to distinguish relaxation from the anticipation of a reward. If the owner reinforces the dog for lying still rather than being relaxed, there is a risk that the dog continues to anticipate rewards rather than also being reinforced through the feeling of relaxation. This may mean that it becomes very hard to get rid of the external rewards. In cue-induced relaxation, the owner can start with some basic knowledge of stress signaling, before moving on to more challenging situations for the dog in which skilful reading of body language becomes more important. It is advisable that the relaxed state of the dog in the proposed starting situation is confirmed by the counsellor using video footage. Third, the owner must be skilful at delivering the reinforcement with the appropriate timing, whether this is by hand or using a remote-controlled device. Since there is no active reinforcement by the owner in the cue-induced relaxation, such an issue does not apply to that technique.

There may be other ways of performing classical conditioning to address social impairment in dogs towards their owners. One way would be to make life entirely predictable for the dog, with identical routines each day and announcing all actions by the owner using the same, specific words each time. In a day-to-day context, however, such rigour is practically unachievable. More likely, the owner will inconsistently use the predictive verbal cues and will at times deviate from daily routines. Since the contingency would not be perfect, actions by the owner would be poorly predicted [60], in which case the emotional response of the dog is expected to worsen rather than improve, but systematic research is needed to confirm this. However, also during the desensitisation process using cue-induced relaxation, the owner must ensure his/her behaviour is 100% of the time conducive for that emotion during the time the safety cue is visible to the dog. If not, the cue could be “poisoned”, in that an undesirable negative significance is attributed to the safety cue, thereby rendering it very difficult to be used as a relaxation cue [61]. A second pitfall is that the conditioning of the cue must be tested and, if it the association is not there yet, there is a risk of poisoning the cue then, too. A third pitfall of cue-induced relaxation is that the owners may conclude that the training does not work because they see nothing happening (the dog does nothing), while, in fact, it is an important objective. This apparent lack of progress may cause owners to abandon the training too early, as the first author has already observed in the past. It is therefore important to explain to the owners what to expect of the training and to emphasise the importance of regular repetition.

The protocol we proposed uses only desensitization without counterconditioning. When using counterconditioning, the purpose is to associate a positive emotion by giving something nice to the dog (e.g., food or attention) when the stimuli are presented during systematic desensitization. The combination of both has been known to work well for dogs with other behaviour problems, such as anxiety towards strangers and other dogs. [27,31,35]. Research in dogs and other species has revealed, however, that desensitization alone is also and sometimes even more effective [55,62]. Other research even mentioned the risk of ineffectiveness of the combination of desensitization and counterconditioning [27]. In case of dogs with social impairment towards their owners, common counterconditioning stimuli such as treats that are given or physical contact such as petting are counter-indicated, for the same reason as we described above. The first author has observed remarkable changes in dogs using cue-induced relaxation. It is likely that the relaxation the dog experiences in itself is reinforcing and that, in that sense, there is an aspect of counterconditioning present in the proposed protocol.

To maximise the effect of the safety signal it is important to perform the procedure of desensitisation in different contexts [63]. Having more contexts in which the dog feels comfortable contributes to overall stress reduction and facilitates habituation to other trivial actions by the owner. Owing to this and the fact that very difficult situations that the dog cannot handle yet are temporarily avoided, the dog will experience an increasing number of situations in which he is comfortable in the presence of the owner. From that point and with the other advantages of the treatment program (knowledge of the owner about dog language and the vulnerability of their dog) a relationship built on predictability can be installed. As the owner becomes more capable of detecting the early signs of stress and recognising which stimuli (and at what intensity) the dog will react to, the more the owner is capable to create a safe environment from the point of view of the dog. As a result, the dog experiences that the owner is the key to feeling safe, relaxed and even content, which is the basis for further positive evolution.

One of the changes that was made to the BMP based on these cases is that, for an owner that is less capable of reading dog body language, it is better not to search too long for existing daily situations that the dog finds relaxing. Instead, it is better to immediately switch to creating such a new situation and give specific instructions to the owner not to move, rather than continuing to search for an existing situation in daily life. On the other hand, the situations where the owner identified the dog as being relaxed while he was not will already prove to be excellent teaching material to give the owner more insight into stress signaling. Finally, for dogs that become too aroused when food is involved during the predictability game, it is better not to perform this game, but to immediately proceed to the presentation of the safety cue. It is also important to note that the BMP can only be successful if all owners are on board. If only one owner is meticulous about executing the BMP and the dog remains uncomfortable around other owners, there is a high likelihood that the treatment will fail. Finally, as we did not take this into account now, it would also be useful to examine whether olfactory signals, originating from the emotional state of the owner when carrying out the BMP, may present a confounding factor in the treatment [64].

## 5. Conclusions

In this paper, we have described a behaviour modification plan to treat dogs with impaired social functioning in relation to their owners. The cases we have presented illustrate the possibilities of this technique, but systematic research is needed into the cause–effect relationship of the whole plan and in particular the different aspects of the plan. It is important to note that there are different parts and that, at this point, it is not known what the relative contribution of each part of the programme is to the improvements that were observed in the dogs we have presented cases for.

## Figures and Tables

**Table 1:** Breed, gender, neutering status, origin, age at first visit to the counsellor, and living conditions of five dogs with social impairment towards their owners.

Dog	Breed	Gender	Neutered	Origin *	Age at First Visit (Months)	Number of Family Members in Household
A	Labrador retriever	M	No	1	14	2
B	Labrador retriever	M	No	1	14	2
C	Malinois	F	No	1	7	2
D	Border collie	M	Yes	4	64	2
E	Shiba Inu	M	No	3	10	3

* 1: occasional breeder who breeds less than 3 L/year; 2: hobby breeder who breeds less than 5 litters/year and has less than 3 different breeds or crossbreeds; 3: professional breeder; 4: breeder–merchant who breeds and also sells puppies from other breeders.

**Table 2:** Timing of BMP (number of sessions and duration) for different acspects, as well as identification of the starting situation. * including the first session which involved also the behaviour assessment (duration 120 min, following sessions 45 min). ** days between the first session and the mentioned session. “O” = owner.

Dog	Total Time of BMP in Months (Number of Sessions *)	Session in Which Relaxing Starting Situation was Identified (Days) **	Relaxing Starting Situation	Time (Days) Until Verification of the Safety Cue Conditioning for the Starting Situation (Session nr)
A	14 (5)	1 (-)	O work on computer	70 (2)
B	3 (4)	1 (-)	Sitting and reading (kitchen)	24 (2)
C	14 (7)	1 (-)	O work on computer	70 (2)
D	24 (10)	3 (79 **)	Sitting at the table	54 (4)
E	27 (13)	1 (-)	Sitting and reading (sofa)	10 (2)
